# Clinical Outcome in Acute Small Bowel Obstruction after Surgical or Conservative Management

**DOI:** 10.1007/s00268-014-2733-6

**Published:** 2014-08-22

**Authors:** Raphael P. H. Meier, Wassila Oulhaci de Saussure, Lorenzo A. Orci, Eveline M. Gutzwiller, Philippe Morel, Frédéric Ris, Frank Schwenter

**Affiliations:** 1 Visceral and Transplant Surgery, Department of Surgery, Geneva University Hospitals and Medical School, Rue Gabrielle-Perret-Gentil 4, 1211 Geneva, Switzerland; 2 Surgical Oncology, Department of Surgery, Montreal University Hospital CHUM, Université de Montréal, 3840 St-Urbain, Montreal, H2W 1T8 Quebec Canada

## Abstract

**Background:**

Small bowel obstruction (SBO) is characterized by a high rate of recurrence. In the present study, we aimed to compare the outcomes of patients managed either by conservative treatment or surgical operation for an episode of SBO.

**Methods:**

The outcomes of all patients hospitalized at a single center for acute SBO between 2004 and 2007 were assessed. The occurrence of recurrent hospitalization, surgery, SBO symptoms at home, and mortality was determined.

**Results:**

Among 221 patients admitted with SBO, 136 underwent a surgical procedure (surgical group) and 85 were managed conservatively (conservative group). Baseline characteristics were similar between treatment groups. The median follow-up time (interquartile range) was 4.7 (3.7–5.8) years. Nineteen patients (14.0 %) of the surgical group were hospitalized for recurrent SBO versus 25 (29.4 %) of the conservative group [hazard ratio (HR), 0.5; 95 % CI, 0.3–0.9]. The need for a surgical management of a new SBO episode was similar between the two groups, ten patients (7.4 %) in the surgical group and six patients (7.1 %) in the conservative group (HR, 1.1; 95 % CI, 0.4–3.1). Five-year mortality from the date of hospital discharge was not significantly different between the two groups (age- and sex-adjusted HR, 1.1; 95 % CI, 0.6–2.1). A follow-up evaluation was obtained for 130 patients. Among them, 24 patients (34.8 %) of the surgical group and 35 patients (57.4 %) of the conservative group had recurrent SBO symptoms (odds ratio, 0.4; 95 % CI, 0.2–0.8).

**Conclusions:**

The recurrence of SBO symptoms and new hospitalizations were significantly lower after surgical management of SBO compared with conservative treatment.

**Electronic supplementary material:**

The online version of this article (doi:10.1007/s00268-014-2733-6) contains supplementary material, which is available to authorized users.

## Introduction

Acute small bowel obstruction (SBO) accounts for about 20 % of all surgical emergencies in Western societies [1]. Small bowel obstruction is caused mainly by postoperative adhesions (more than 75 % of all cases) [2–6]. The operative procedures usually associated with SBO are colectomy, hysterectomy, and appendectomy [7]. Other causes of SBO are Crohn’s disease (7 %), neoplasm (5–10 %), hernia (2 %), or radiation-induced enteritis (1 %) [2–5]. In a series of 29,790 patients with a single previous abdominal or pelvic surgery, Ellis et al. reported that within the following 10 years 34.6 % of them were readmitted with a mean of 2.1 times for a disease related to adhesions [8]. About 10 % of patients have “spontaneous” SBO with no previous abdominal surgery [5]. The management of SBO is based on clinical evaluation, biological tests, and computed tomography (CT) imaging. Conservative treatment with bowel rest, nasogastric tube decompression, and fluid resuscitation is successful in a variable proportion of patients [9]. In conservative management, regular reassessment is mandatory for early recognition of signs of bowel ischemia that would require a surgical operation. Patients with clinical degradation or with a CT scan evoking strangulated SBO need urgent surgery [10, 11]. In a significant proportion of patients both therapeutic options are valid; the choice of the treatment depends mainly on the clinician’s assessment, and therefore it represents a common clinical challenge. On the one hand, successful conservative treatment may leave adhesions that could cause recurrence; on the other hand, surgery may be the source of new adhesions like any other abdominal surgery [12, 13]. Landercasper et al. reported a 42 % risk of SBO recurrence by 10 years, which is lessened in surgically treated patients compared to their conservatively treated counterparts [14]. Another retrospective study analyzed the pattern of recurrence after one or more episodes of SBO [15]; the rate of SBO recurrence was shown to increase proportionally to the number of previous SBO episodes.

In the present study, we report a long-term follow-up of patients treated with surgical versus conservative approach for SBO. The aim was to compare the rate of SBO recurrence, resulting in new hospitalization with or without surgery, as well as the occurrence of SBO symptoms, such as recurrent abdominal pain, at home.

## Patients and methods

### Study design and population

This is a retrospective single-center study of patients hospitalized at University Hospitals of Geneva with acute adhesive SBO between January 2004 and December 2007. The study was approved by the local research ethics committee (protocol NAC 10–015). During the study period, all consecutive patients admitted in our center with an episode of SBO were included in the study. An SBO episode was defined using the code K56.5 from the International Classification of Diseases, 10th Revision (ICD-10). The first hospitalization for SBO occurring during the study period was defined as the index date, and hereafter we refer to the “index SBO episode” (and respectively, “index hospitalization” or “index surgery”). Management included either a standard conservative treatment with intravenous fluid infusion, analgesics and nasogastric tube; or surgical treatment including exploratory laparotomy with adhesiolysis, with or without small bowel resection. Exclusion criteria were large bowel obstruction, incarcerated abdominal wall hernia, early postoperative SBO (within 1 month), inflammatory bowel disease, radiation-induced intestinal fibrosis, and peritoneal carcinomatosis.

### Study variables

The computerized records of the included patients were reviewed for complete information regarding their past surgical history and clinical data about the index SBO episode. Small bowel obstruction severity score was calculated as described in Schwenter et al. [16]. Rehospitalization(s) caused by recurrent SBO and the nature of treatment (surgical vs. conservative) were retrieved from institutional computerized records for every patient. Non-readmitted patients were contacted by phone in order to assess any recurrence of SBO symptoms. Vital status was obtained from the state population office of statistics. Patients were followed up until May 2011.

### Statistical analysis

Differences between groups were analyzed with the *t* test for continuous variables and the Chi square test for binary and categorical variables. Survival analyses were performed with the Kaplan–Meier method and the log rank test. Uni-/multivariate Cox proportional-hazards regression was used to compute hazard ratios (HR). Ninety-five percent confidence intervals (95 % CI) were reported, and an exact two-sided *p* value <0.05 was considered statistically significant.

## Results

### Patient characteristics

During the study period, 221 patients were hospitalized for an index SBO episode. The mean age of patients was 67 (±18) years, and there were 131 women (59.3 %). Patients were categorized into two groups according to their index treatment. One hundred thirty-six patients (61.5 %) underwent operation (surgical group) and 85 patients (38.5 %) were managed with medical treatment (conservative group). Age and sex were equally distributed in the two groups (Table 1). One hundred twenty-one patients (89.0 %) from the surgical group and 80 patients (94.1 %) from the conservative group had had one or more previous abdominal operations (*p* = 0.234). The numbers and types of previous abdominal operations were similar in the two groups. Eighteen patients (13.2 %) from the surgical group and 19 patients (22.4 %) from the conservative group had had one or more previous SBO episode (*p* = 0.096); the type of the management of the previous SBO episode(s) (surgical vs. conservative) was similar for the two groups (a patient was considered as surgically treated when at least one operation was reported in the past surgical history). At the time of index hospitalization, clinical severity score was calculated as follows (one point was given for each present item): pain duration ≥4 days, abdominal guarding, leukocyte ≥10 × 10^9^/l, C-reactive protein ≥75 mg/l, free fluid ≥500 ml on CT scan, reduced contrast enhancement on CT scan; (min–max: 0–6) [16]. As expected, the clinical severity score was higher in the surgical group than in the conservative group (*p* < 0.001). Forty-nine patients (36.0 %) had a score ≥3 in the surgical group, compared to seven patients (8.2 %) in the conservative group. Decision making for surgical management is given in Table 1. Among the ten patients who underwent laparotomy with a severity score of 0, seven had a transition zone on CT scan, two underwent operation because of a clinical degradation, and one had small bowel dilatation >4.5 cm on CT scan. In the surgical group, 17 patients (12.5 %) had a delayed surgery (i.e., beyond 24 h after hospital admission) and 44 (32.4 %) required bowel resection. Patients in the surgical group had a significantly longer hospital stay than patients in the conservative group—12.0 (±8.5) versus 6.6 (±3.6) days, respectively (*p* < 0.001). Nine patients (6.6 %) died during the postoperative course of the index SBO surgery; patient characteristics and cause of death are available in Supplemental Table S1 that can be found online at electronic supplementary material section. In these nine patients, the mean age was 77 (±17) years, and the most common cause of death was postoperative pulmonary distress. No death was reported for the conservative group during the 30 days following the index hospital stay.

**Table 1:** Patient characteristics

Variable	Surgical management (*n* = 136)	Conservative management (*n* = 85)	*p* value^a^
Age, years (mean ± SD)	68.4 ± 18.4	64.7 ± 16.5	0.133
Sex, male (%)	52 (38.2)	38 (44.7)	0.399
Previous abdominal operation (%)	121 (89.0)	80 (94.1)	0.234
Patients with previous surgery (%)			
0 abdominal operation	15 (11.0)	5 (5.9)	0.120
1 abdominal operation	56 (41.2)	28 (32.9)	
2 or more abdominal operations	65 (47.8)	52 (61.2)	
Previous appendectomy (%)	63 (46.3)	41 (48.2)	0.784
Previous cholecystectomy (%)	21 (15.4)	14 (16.5)	0.852
Previous colorectal surgery (%)	25 (18.4)	24 (28.2)	0.097
Previous gynecologic surgery (%)	26 (19.1)	21 (24.7)	0.398
Previous urologic surgery (%)	8 (5.9)	7 (8.2)	0.585
Previous hernia repair (%)	18 (13.2)	7 (8.2)	0.283
Previous gastric surgery (%)	11 (8.1)	4 (4.7)	0.417
Previous small bowel surgery (%)	5 (3.7)	6 (7.1)	0.342
Previous vascular surgery (%)	2 (1.5)	3 (3.5)	0.375
Previous operation for cancer (%)	22 (16.2)	12 (14.1)	0.707
Previous SBO episode(s) managed surgically (%)	4 (2.9)	4 (4.7)	0.488
Previous SBO episode(s) managed conservatively (%)	14 (10.3)	15 (17.6)	0.151
Clinical severity score (%)^b^			
0	10 (7.4)	15 (17.6)	<0.001
1	31 (22.8)	30 (35.3)	
2	46 (33.8)	33 (38.8)	
3	38 (27.9)	6 (7.1)	
4	10 (7.4)	1 (1.2)	
5	1 (0.7)	0 (0.0)	
6	0 (0.0)	0 (0.0)	
Decision making for surgical management (%)			
CT transition zone	83 (61.0)	NA	NA
Clinical degradation^c^	23 (16.9)		
CT: volvulus	18 (13.2)		
CT: reduced contrast enhancement	8 (5.9)		
Other^d^	4 (2.9)		
Delay before surgery ≥24 h (%)	17 (12.5)	NA	NA
Small bowel resection (%)	44 (32.4)	NA	NA
Length of index hospital stay, days (mean ± SD)	12.0 ± 8.5	6.6 ± 3.6	<0.001
Death during the index hospital stay (%)^e^	9 (6.6)	0 (0.0)	0.014

Recorded during the index hospitalization for a SBO episode

*SD* standard deviation, *NA* not applicable, *SBO* small bowel obstruction

^a^Student’s *t* test for continuous variables, χ^2^ test for binary or categorical variable (global *p* value)

^b^Calculated as described by Schwenter et al. 2010. One point was given for each present feature: pain duration ≥4 days, abdominal guarding, leukocyte count ≥10 × 10^9^/l, C-reactive protein ≥75 mg/l, free fluid ≥500 ml on CT scan, reduced contrast enhancement on CT scan. The score ranged from 0 to 6

^c^Development of peritonism, no flatus after 24 h of observation, or hemodynamic instability

^d^Includes diffuse small bowel feces sign (*n* = 1), small bowel dilatation >4.5 cm (*n* = 1), SB wall major inflammation (*n* = 2)

^e^Death occurring ≤30 days after SBO surgery or conservative management initiation

### Comparison of readmission and operation hazard ratios associated with management (surgical vs. conservative)

The median follow-up time (interquartile range) for the whole study population was 4.7 (3.7–5.8) years. Nineteen patients (14.0 %) from the surgical group were rehospitalized for a new SBO episode, as were 25 patients (29.4 %) from the conservative group (Table 2). Among the 19 patients who had postoperative SBO, 2 had SBO recurrence within the 30-day postoperative period (both underwent reoperation), and 8 others had SBO recurrence within 1 year following the index operation (5 underwent reoperation). The mean interval between the first hospitalization and rehospitalization was 1.1 (±1.1) years in the surgery group and 1.7 (±1.4) years in the conservative group (*p* = 0.121). Four patients (2.9 %) in the surgical group were readmitted twice or more, whereas seven patients (8.2 %) in the conservative group required more than one readmission. Among those initially assigned to the surgical group, ten patients (7.4 %) needed a surgical treatment for recurrent SBO compared with six patients (7.1 %) in the conservative group. The need for a bowel resection during surgery for recurrent SBO was non-significantly lower in the surgery group compared to the conservative group. Seven patients from the surgical group required reoperation within 1 year, two of them during the 30-day postoperative period. The first of those two patients was reoperated for persistence of clinical signs of SBO, and during that operation the surgeon found an extensive area of necrosis of the small bowel that needed resection. The second patient had a good initial evolution, but 2 weeks postoperatively a clinical degradation developed, with abdominal pain, elevated markers of inflammation, and a CT scan showing a segmental ischemic small bowel and free fluid; operation revealed a segmental small bowel necrosis with perforation, and this segment was resected. The remaining five patients underwent reoperation 50, 177, 184, 230, and 351 days after the index operation. Extensive adhesions were found in four of them; in one patient who had a surgical history of gastric bypass, mesenteric windows were closed. None of the patients required a small bowel resection.

**Table 2:** Hospitalization and operation for SBO recurrence, and overall mortality associated with index management (surgical vs. conservative)

Variable	Surgical management (*n* = 136)	Conservative management (*n* = 85)	Unadjusted HR for surgery (95 % CI)	*p* value	Adjusted HR for surgery (95 % CI)^a^	*p* value
Hospitalization for recurrent SBO (%)	19 (14.0)	25 (29.4)	0.5 (0.3–0.9)	0.016	0.5 (0.3–0.9)	0.014
>1 hospitalization for recurrent SBO (%)	4 (2.9)	7 (8.2)	0.3 (0.1–1.1)	0.063	0.3 (0.1–1.1)	0.066
Patients operated for SBO recurrence (%)	10 (7.4)	6 (7.1)	1.1 (0.4–3.1)	0.824	1.1 (0.4–3.0)	0.852
Small bowel resection (%)	3 (2.2)	3 (3.5)	0.7 (0.1–3.3)	0.610	0.6 (0.1–2.9)	0.489
>1 operation for recurrent SBO (%)	2 (1.5)	1 (1.2)	1.2 (0.1–13.5)	0.870	1.2 (0.1–13.5)	0.869
Death until end of follow-up (%)	44 (32.4)	15 (17.6)	2.0 (1.1–3.6)	0.023	1.5 (0.8–2.7)	0.196

Hazard ratios (HR) and *p* values were calculated with univariate and multivariate Cox proportional-hazards regression analysis

^a^Age- and sex-adjusted

**Fig. 1:**
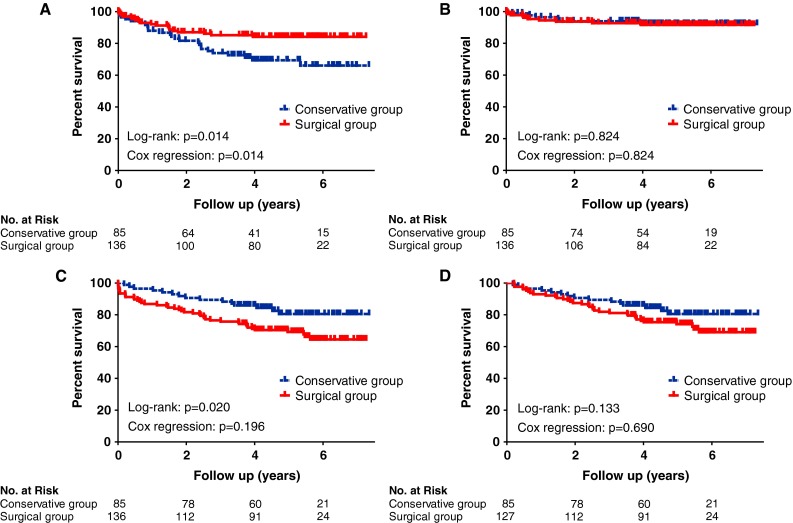
Kaplan–Meier estimates of **a** the hospitalization-free survival, **b** operation-free survival, **c** overall survival, and **d** survival from hospital discharge (i.e., excluding patients who died within 30 days after admission). *p* values were calculated with the log rank test. Age- and sex-adjusted *p* values were calculated with multivariate Cox proportional-hazards regression analysis

Two patients (1.5 %) in the surgical group and one (1.2 %) in the conservative group required operation to treat further SBO recurrence. Overall, 44 patients (32.4 %) died in the surgical group and 15 (17.6 %) in the conservative group. The age- and sex-adjusted HR for surgery was 1.5; 95 % CI, 0.8–2.7; *p* = 0.196. When considering only death occurring more than 30 days after SBO surgery or conservative management initiation (i.e., excluding postoperative death), the age- and sex-adjusted HR (95 % CI) was 1.1 (0.6–2.1); *p* = 0.690. Kaplan–Meier estimates of the hospitalization-free survival, operation-free survival, overall survival, and survival from the date of hospital discharge (>30 days after admission) are shown in Fig. 1.

### Comparison of small bowel obstruction symptoms between surgical and conservative groups

A follow-up evaluation was obtained for 130 patients [80.2 % of the patients still alive (130/162)]. The remaining patients had invalid or missing contact information (*n* = 32). Comparing the surgical group versus the conservative group, we found that 24 patients (34.8 %) in the surgical group had SBO symptom recurrence, versus 35 patients (57.4 %) in the conservative group (Table 3). Almost all symptomatic patients reported that they suffered from abdominal pain. Nausea and vomiting were reported by 25.4 % of the symptomatic patients (15/59). The delay to SBO symptom recurrence following the index hospitalization did not differ significantly between the surgical group and the conservative group [mean (± SD) 1.1 (0.9.)] year in both groups; *p* = 0.925). In symptomatic patients, the median frequency of abdominal pain episodes was one episode per week (range: 0–14); nausea and vomiting were reported to occur twice a week (range: 1–14).

**Table 3:** Recurrence of SBO symptoms, univariate logistic regression

Variable	Surgical management (*n* = 69)	Conservative management (*n* = 61)	OR (95 % CI)	*p* value
Recurrence of SBO symptoms (any) (%)	24 (34.8)	35 (57.4)	0.4 (0.2–0.8)	0.011
Abdominal pain (%)	23 (33.3)	34 (55.7)	0.4 (0.2–0.8)	0.011
Nausea and vomiting (%)	7 (10.1)	8 (13.1)	0.7 (0.3–2.2)	0.598

Odds ratios (OR) and *p* values were calculated using univariate logistic regression analysis

### Comparison between patients operated after 24 h of admission and those operated within 24 h for an index SBO episode

Patients operated beyond 24 h after admission for an SBO episode had a non-significantly lower rehospitalization rate and similar overall mortality, compared with those who were operated within 24 h of admission (Supplemental Table S2, online electronic supplementary material). Reoperation for small bowel obstruction recurrence was not observed in patients operated 24 h beyond their admission. Ten patients in the <24 h group underwent reoperation. Symptoms of SBO occurrence (considering patients who responded to follow-up evaluation) was similar between the two groups [25.0 % (2/8) vs. 36.1 % (22/61), OR, 0.6; 95 % CI, 0.1–3.2; *p* = 0.540].

### Comparison between patients with and without small bowel resection during the index SBO episode management

Patients who underwent a small bowel resection during the index SBO episode had a similar rehospitalization rate and a nonsignificantly higher operation rate for small bowel obstruction recurrence, as well as similar overall mortality, compared to those who had no resection (Supplemental Table S3, online electronic supplementary material). Symptoms of SBO occurrence (considering patients who responded to follow-up evaluation) were similar between the two groups [35.0 % (7/20) vs. 34.7 % (17/49), OR, 1.0; 95 % CI, 0.3–3.0; *p* = 0.981].

## Discussion

In the present study we compared the outcomes of patients treated either by surgical or conservative management for an SBO episode. The main findings are that patients treated by surgery are 50 % less likely to require rehospitalization and 60 % less likely to suffer from “everyday” SBO symptoms at home compared with those managed by a conservative approach. Furthermore, surgery for SBO does not significantly modify the rate of a subsequent SBO operation.

Our results concerning SBO recurrence are in accordance with those of Landercasper et al., who reported a statistically significant difference in rehospitalization rates between patients treated surgically (21 %) or conservatively (38 %) (*p* = 0.001) [14]. They observed operation rates for a new SBO episode of 10 and 17 %, respectively (*p* = 0.08). Of note, patients with SBO episodes caused by malignancy and inflammatory bowel disease were also included by Landerscaper et al.; this may explain the higher recurrence rates compared to our study. Fevang et al. reported that their patients who had surgery were 45 % less likely to suffer from recurrent SBO than patients managed conservatively (relative risk, 0.55; 95 % CI, 0.35–0.86) [15]. They also reported that the risk of being operated for a new SBO episode is the same regardless of the initial treatment (relative risk, 0.79; 95 % CI, 0.39–1.59). Our results regarding morbidity of SBO patients according to the type of treatment received are consistent with previous studies [14, 17–20]. Concerning the postoperative mortality, a 6.6 % 30-day mortality rate was observed in our study, which is in accordance with other reports [4, 18, 19]. Overall, more patients died in the surgical group than in the conservative group. However, the modality of treatment seemed not to be involved in this difference. Indeed, the patients in the surgical group were slightly older that patients treated conservatively, and once adjusted for age and sex, overall mortality was not different between the two groups. Moreover, only the sickest of patients died during the postoperative period.

The assessment of SBO symptoms occurrence obtained by phone calls showed that conservatively treated patients had a significantly higher rate of SBO symptoms in their everyday life than surgically treated ones. This result is of importance for the decision-making process regarding the optimal treatment, as it indicates that patients who underwent surgical management have decreased risks of recurrence. Fevang et al. reported that patients treated for SBO were more prone to experiencing abdominal pain than the normal subjects (especially women and those with matted adhesions). However, to our best knowledge, our study is the first to show that patients treated with surgery for SBO had 60 % less frequent subsequent SBO symptoms at home compared to patients with conservative treatment.

The benefit of surgical treatment observed in our study has to be balanced with the risks associated with surgery, particularly for patients with comorbidities and advanced age. Moreover, laparoscopic adhesiolysis for chronic abdominal pain seems not to be effective [21]. Thus, an individualized treatment should be considered, based on the severity of the SBO episode as well as the general medical condition of the patient. A prospective study combining a score of severity of SBO [16] and a score indicating patient medical condition (APACHE II score [22] ) could help in the decision for taking either a conservative or a surgical approach.

Although it is commonly accepted that early operation for SBO (<24 h) decreases immediate morbidity [20, 23], no difference in the long-term outcomes between patients operated within or after 24 h of admission were found in our study. Based on this observation, it seems reasonable to attempt conservative management for patients without any signs of severity. Surgical treatment would be recommended in the absence of bowel function recovery within 24 h after an oral water-soluble contrast agent test [24].

A limitation of the present study is the selection bias regarding assignment of patients to study groups. Patients in the conservative group had lower severity scores. However, it seems unlikely that this bias affects the long-term outcomes. Moreover, the patient groups were similar regarding major causes for SBO, i.e., colectomy, hysterectomy, and appendectomy, as compared to historical series [7]. A randomized controlled trial would be informative to definitively determine whether surgery is actually advantageous in terms of recurrence of SBO in non-strangulated SBO patients. However, such a study would expose patients with no signs of severity to the risks of a surgical operation and is ethically questionable. Moreover, a certain amount of bias in a randomized study comparing conservative and surgical management seems unavoidable. Another fact that would limit the feasibility of such a study is the inability to determine with confidence whether the small bowel is strangulated or not, which would restrict the investigators from obtaining a homogeneous group with non-strangulated SBO only.

In conclusion, this study indicates that patients undergoing surgical management for SBO had reduced risks of recurrence requiring hospitalization, as well as SBO symptoms as compared to those with conservative treatment. Based on these findings, the following clinical recommendation for the management of small bowel obstruction may be considered: patients with three or more of the following criteria (pain duration ≥4 days, abdominal guarding, leukocyte ≥10 × 10^9^/l, C-reactive protein ≥75 mg/l, free fluid ≥500 ml on CT scan, or reduced contrast enhancement on CT scan) should undergo prompt surgical intervention as it allows both obstruction removal and long-term reduction of recurrent SBO episodes. The decision to operate should also take into account the evolution of the clinical status and laboratory values, additional CT findings (e.g., volvulus, transition zone, reduced contrast enhancement, small bowel feces sign), as well as the patient’s general condition, comorbidities, and surgical history.

## Electronic supplementary material

Below is the link to the electronic supplementary material.

Supplementary material 1 (DOC 46 kb)

Supplementary material 2 (DOC 41 kb)

Supplementary material 3 (DOC 39 kb)
