# Supplementary material for: Clinical Outcome in Acute Small Bowel Obstruction after Surgical or Conservative Management
Source: World J Surg. 2014 Aug 22;38(12):3082–8. doi: 10.1007/s00268-014-2733-6 (PMC4232739; doi:10.1007/s00268-014-2733-6)
Supplement: Supplementary file 1 — Supplementary material 1 (DOC 46 kb) [file 268_2014_2733_MOESM1_ESM.doc]

**Supplemental table S1.** Characteristics and cause of death of the patients who died within 30 days after SBO operation.

| Patients | Age (years) | Sex | Number of previous abdominal operation(s) | Previous SBO episode | Decision making for surgical management | Clinical severity score ^a^ | Delay before surgery ≥24h | Small bowel resection | Death occurred on POD | Cause of death |
| --- | --- | --- | --- | --- | --- | --- | --- | --- | --- | --- |
| Patient 1 | 74 | F | 0 | No | Clinical degradation | 3 | No | Yes | 2 | Surgery revealed an extensive necrosis of the small bowel, patients and family refused further aggressive management |
| Patient 2 | 82 | M | 1 | No | CT : reduced contrast enhancement | 3 | No | Yes | 14 | Postoperative respiratory distress caused by massive pleural effusion, complicated by ascites and rapidly progressive renal insufficiency |
| Patient 3 | 92 | F | 0 | No | Clinical degradation | 1 | No | No | 2 | Septic choc two days after surgery followed by malignant cardiac arrhythmia |
| Patient 4 | 85 | F | 1 | No | CT : transition zone | 2 | Yes | No | 1 | Peroperative respiratory distress caused by massive bronchoaspiration during the induction of anesthesia |
| Patient 5 | 84 | F | 1 | No | CT : volvulus | 4 | No | No | 3 | Respiratory distress in a multimorbidity patient with extensive necrosis of the small bowel |
| Patient 6 | 34 | F | 2 | Yes | Clinical degradation | 2 | Yes | Yes | 4 | Postoperative respiratory distress caused by massive bronchoaspiration complicated by ARDS and septic choc |
| Patient 7 | 80 | M | 1 | No | CT : transition zone | 1 | No | Yes | 12 | Postoperative respiratory distress caused by pneumonia in a cachectic patient with COPD |
| Patient 8 | 79 | M | 1 | No | Clinical degradation e | 1 | No | No | 21 | Septic choc in a patient with advanced Alzheimer disease |
| Patient 9 | 79 | F | 1 | No | CT : transition zone | 4 | No | Yes | 17 | Hypoxic and hypercapnic respiratory failure in a patient with COPD |

SBO: small bowel obstruction, COPD: Chronic obstructive pulmonary disease, ARDS: Acute respiratory distress syndrome

^a^ Calculated according to Schwenter et al. 2010
