# Supplementary material for: Clinical Outcome in Acute Small Bowel Obstruction after Surgical or Conservative Management
Source: World J Surg. 2014 Aug 22;38(12):3082–8. doi: 10.1007/s00268-014-2733-6 (PMC4232739; doi:10.1007/s00268-014-2733-6)
Supplement: Supplementary file 2 — Supplementary material 2 (DOC 41 kb) [file 268_2014_2733_MOESM2_ESM.doc]

**Supplemental table S2.** Hospitalization and operation for small bowel obstruction recurrence, and overall mortality associated with delay before the index operation for small bowel obstruction (performed before or after 24 hours of admission). Hazard ratios and p values were calculated using uni- and multivariate Cox proportional-hazards regression analysis.

| Variable | Delay before surgery ≥24h (n=17) | Delay before surgery <24h (n=119) | Unadjusted HR for ≥24h delay  (95% CI) | p value | Adjusted HR for ≥24h delay  (95% CI) ^a^ | p value |
| --- | --- | --- | --- | --- | --- | --- |
| Hospitalization for recurrent SBO (%) | 1 (5.9) | 18 (15.1) | 0.4 (0.5 – 2.9) | 0.357 | 0.4 (0.5 – 2.9) | 0.361 |
| Patients operated for SBO recurrence (%) | 0 (0.0) | 10 (8.4) | NA | NA | NA | NA |
| Death until end of the follow up (%) | 6 (35.3) | 38 (31.9) | 1.1 (0.5 - 2.6) | 0.803 | 1.1 (0.4 - 2.5) | 0.889 |

SBO: small bowel obstruction, HR: hazard ratio, NA: not applicable

^a^ Age- and sex-adjusted
