# Supplementary material for: Clinical Outcome in Acute Small Bowel Obstruction after Surgical or Conservative Management
Source: World J Surg. 2014 Aug 22;38(12):3082–8. doi: 10.1007/s00268-014-2733-6 (PMC4232739; doi:10.1007/s00268-014-2733-6)
Supplement: Supplementary file 3 — Supplementary material 3 (DOC 39 kb) [file 268_2014_2733_MOESM3_ESM.doc]

**Supplemental table S3.** Hospitalization and operation for small bowel obstruction recurrence, and overall mortality associated with the type of surgery during the index small bowel obstruction management (with or without small bowel resection). Hazard ratios and p values were calculated using uni- and multivariate Cox proportional-hazards regression analysis.

| Variable | Small bowel resection (n=44) | No resection (n=92) | Unadjusted HR for resection  (95% CI) | p value | Adjusted HR for resection  (95% CI) ^a^ | p value |
| --- | --- | --- | --- | --- | --- | --- |
| Hospitalization for recurrent SBO (%) | 6 (13.6) | 13 (14.1) | 1.0 (0.4 - 2.6) | 0.982 | 0.9 (0.4 - 2.5) | 0.889 |
| Patients operated for SBO recurrence (%) | 4 (9.1) | 6 (6.5) | 1.4 (0.4 - 5.0) | 0.601 | 1.3 (0.4 - 4.7) | 0.677 |
| Death until end of the follow up (%) | 14 (31.8) | 30 (32.6) | 1.0 (0.5 - 1.9) | 0.993 | 0.9 (0.5 - 1.7) | 0.780 |

SBO: small bowel obstruction, HR: hazard ratio, NA: not applicable

^a^ Age- and sex-adjusted
